# Growth, Physiology and Nutritional Quality of C_4_ Halophyte *Portulaca oleracea* L. Grown Aeroponically in Different Percentages of Artificial Seawater under Different Light-Emitting Diode Spectral Qualities

**DOI:** 10.3390/plants12183214

**Published:** 2023-09-08

**Authors:** Jie He, Su Yee Leng, Lin Qin

**Affiliations:** Natural Sciences and Science Education Academic Group, National Institute of Education, Nanyang Technological University, 1 Nanyang Walk, Singapore 637616, Singapore; suyeeleng@gmail.com (S.Y.L.); lin.qin@nie.edu.sg (L.Q.)

**Keywords:** C_4_ halophyte, LED light qualities, light use efficiency, nitrogen metabolism, nutritional quality, yield

## Abstract

Edible halophyte *Portulaca oleracea* L., known as purslane, was grown in two percentages of artificial seawater (ASW) under two combined red (R) and blue (B) LED spectra. High salinity (40% ASW) negatively affected shoot productivity and leaf growth of purslane compared to those grown in 10% ASW. Photosynthetic pigment and total reduced nitrogen concentrations were significantly higher in purslane grown in 10% ASW than in 40% ASW. However, LED spectral quality did not markedly influence these parameters. Grown in 10% ASW under R/B 2.2, purslane had the highest maximum nitrate reductase activity, while those in 40% ASW under R/B 2.2 had the highest activation state. Under both light qualities, purslane had a sevenfold increase in proline concentration in 40% ASW than in 10% ASW. Total phenolic compounds’ concentration was the highest in 10% ASW under R/B 0.9, while there were no significant differences in the accumulation of total soluble sugars and ascorbic acids among all plants. Antioxidant enzymes activities were lower in 40% ASW under R/B 2.2 compared to the other conditions. In conclusion, salinity affected the yield, physiology and nutritional quality of purslane. The impacts of LED spectral quality on purslane were only reflected by certain physiological and nutritional parameters.

## 1. Introduction

*Portulaca oleracea* L. (known as purslane) is an annual succulent dicot plant, belonging to the family Portulaceceae Juss [1]. It grows mainly in the tropics and subtropics [1] but it is consumed worldwide [2]. It is an important plant because of its nutritional value [3,4] and medicinal benefits [4,5]. Interest in cultivating purslane as a vegetable crop has increased because it has a high accumulation of phytochemicals such as total phenolic compounds (TPC), ascorbic acid (ASC) and vitamins [4,6,7] together with a high amount of dietary minerals [7,8]. Purslane is a C_4_ halophyte. It is also a facultative crassulacean acid metabolism (CAM) plant, which is able to undergo a switch from the C_4_ to CAM pathway after being subjected to drought [9] or salt stress [7,10] to enhance water use efficiency. It has become an important model crop not only for studying facultative C4/CAM photosynthesis evolution but also for exploring its future role for food security [7,8,9].

Due to its salt tolerance, purslane can be cultivated with saline water. Production of purslane is considered one of the strategies to ensure food security, especially in Singapore, where the biggest threat in food production is fresh water scarcity [8,11]. Our studies showed that it is feasible to grow halophytes such as purslane and ice plants indoors with enhanced productivity and nutritional quality through the optimal selection of salinity and/or nutrient supply [8,12]. Halophytes are defined as plants which grow and complete their life cycle in habitats where the salt content is high [13]. However, tolerance of halophytes to salinity varies among plant species. According to the literature, halophytes can complete their life cycles in soil containing more than 200 mM NaCl, while glycophytes cannot [14]. Purslane is considered a moderately salt-tolerant species, with a capacity to withstand salinity of more than 200 mM NaCl [15,16,17]. We have previously studied the responses of hydroponically grown purslane to different salinities indoors under a red (R) and blue (B) LED ratio of 2.2. It was found that purslane grown with 100 mM NaCl had faster leaf growth and greater biomass accumulation compared to those grown with fresh water, 200 and 300 mM NaCl [8]. High salinity resulted in decreases in shoot production due to leaf water deficit and reduced photosynthetic performance [8].

When a CAM is induced in purslane plants under drought stress, there is a clear metabolic shift, implying the metabolic flexibility of purslane [18]. We found that CAM-induced purslane grown in 300 mM NaCl had the highest leaf proline concentration. Yazici et al. [19] found that increasing NaCl concentration from 0 to 140 mM caused an increase in proline concentration, while the growth of purslane was more inhibited under 140 mM NaCl than under 70 mM NaCl. With the accumulation of proline under high salinity, purslane could enhance the capacity of the antioxidative system to scavenge reactive oxygen species (ROS) supported by suppressed levels of lipid peroxidation and enhanced activities of antioxidant enzymes [19]. Halophytes also accumulate high concentrations of total ASC and TPC under high salinity conditions [20]. However, in our previous study, the concentrations of total ASC and TPC were higher in purslane grown in 100 mM NaCl than in 200 and 300 mM NaCl [8]. In another study with the ice plant by our team, controversial results were obtained [21]. The ice plant had higher accumulations of proline, total soluble sugar, ASC and TPC in plants with 250 and 500 mM NaCl than with 100 mM NaCl [21]. The correlations among the levels of proline, antioxidants and salinity seem to be dependent on species [22] or genotypes [23], cultivation methods and other growth conditions such as light quality [21].

In the study with the ice plant, there was an interaction between the LED ratio and salinity detected for shoot FW and shoot DW, maximal efficiency of PS II photochemistry, non-photochemical quenching (NPQ) and proline content [21]. In the study of purslane microgreens, Giménez et al. [24] reported that yield was enhanced under combined RB light and RB+FR (far red) lights compared to FL (fluorescent lamps) and in salinity compared to non-salinity conditions. Purslane microgreens grown under RB light also had the highest concentration of TPC, while salinity reduced the concentrations of TPC, total chlorophyll and carotenoids. The NO_3_^−^ concentration was also reduced when microgreens were grown under RB and RB+FR compared to FL light [24]. To our knowledge, this is the first study which aimed to investigate the effects of salinity on the nutritional values and physiological performance of purslane under different LED spectral qualities using indoor aeroponic vertical farming systems.

Proline accumulation, which is affected by salinity and nutrient deficiency [24], could be an indicator for unbalanced nitrogen (N) nutrition [25]. The physiological parameters investigated in this study included maximal efficiency of PS II photochemistry (F_v_/F_m_ ratio) and N metabolism such as concentrations of nitrate (NO_3_^−^), total reduced nitrogen (TRN), total soluble protein (TSP), maximum nitrate reductase activities (NRA_max_) and NR activation state (NR_act_). Nitrate reductase (NR) catalyzes the initial step of plant NO_3_^−^ assimilation. NRA_max_ and NR_act_ may be important factors affecting the N acquisition in plants subjected to salinity stress. For nutritional quality, proline, total soluble sugar (TSS), which is also an osmoprotectant, and antioxidants such as total ASC and TPC were studied. There is a lack of understanding if the activities of enzymatic antioxidants such as superoxide dismutase (SOD), catalase (CAT), ascorbate peroxidase (APX) and glutathione reductase (GR) in purslane are correlated with salinity stress and LED quality. Thus, the novelty of this study also involved investigating the activities of these antioxidant enzymes. To achieve our objectives, 10% or 40% artificial seawater (ASW) that simulates seawater were used to grow purslane in our indoor vertical farming system under a combination red (R) and blue (B) LED ratio of 0.9 (R/B 0.9) and 2.2 (R/B 2.2). The findings of the study could provide scientific information on purslane cultivation indoors, which could improve its productivity and nutritional quality.

## 2. Results and Discussion 

### 2.1. Productivity and Leaf Traits

**Figure 1:**
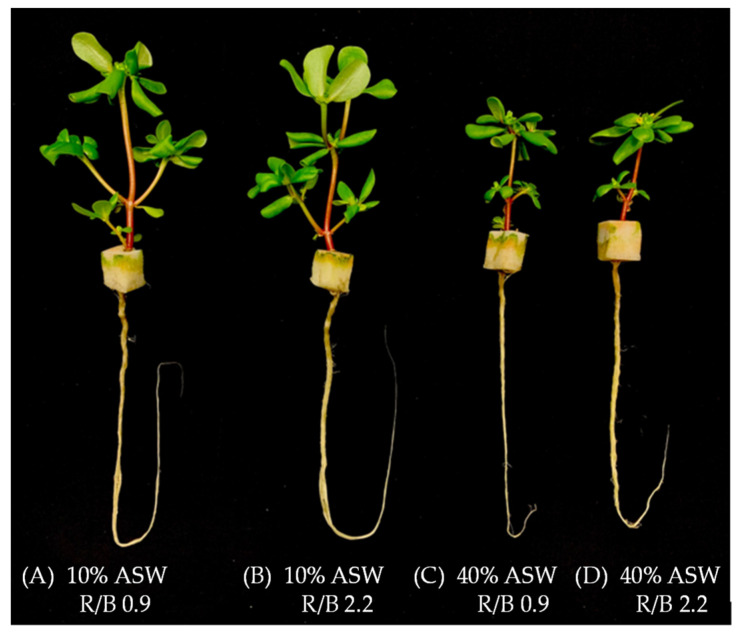
Aeroponically grown purslane in two different percentages of artificial seawater (ASW) under two different LED R/B ratios, 14 days after transplanting.

**Figure 2:**
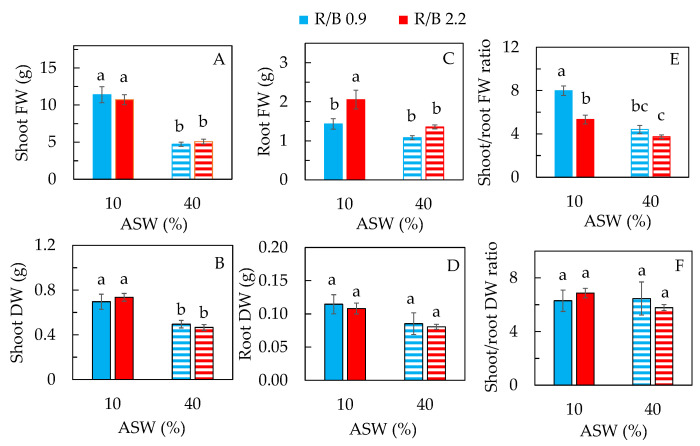
Shoot FW (**A**), shoot DW (**B**), root FW (**C**), root DW (**D**), shoot/root FW ratio (**E**) and shoot/root DW ratio (**F**) of purslane grown in two different percentages of artificial seawater (ASW) under two different LED R/B ratios for 14 days. Values are means ± standard error of 4 different plants. Means with different letters above the column represent significant differences according to Tukey’s multiple comparison test (*p* < 0.05).

Figure 1 shows purslane plants grown in 10% (A, B) and 40% ASW (C, D) under R/B 0.9 (A, C) and R/B 2.2 (B, D), respectively, for 14 days. Although all plants were healthy, those grown in 10% ASW had much larger shoots and roots than in 40% ASW under both R/B ratios. There were no significant differences in shoot sizes between the two R/B ratios grown with the same ASW (Figure 1). However, plants grown under R/B 2.2 seem to have bigger roots, although data are not available. The growth of purslane shown in Figure 1 is supported by the fact that the shoot fresh weight (FW) and dry weight (DW), and the root FW of plants grown in 10% ASW were higher than in 40% ASW (Figure 2A–C) without differences in shoot FW and DW between R/B 0.9 and R/B 2.2 (Figure 2A,B). Similar trends were also recorded for leaf FW and DW and stem FW and DW (Figure S1). However, purslane grown in 10% ASW had significantly higher root FW under R/B 2.2 than under R/B 0.9 (Figure 2C). Although statistically there was no difference in root FW of purslane grown in 40% ASW between R/B ratios, plants grown under R/B 2.2 also had higher values than under R/B 0.9 (Figure 2C).

It was previously reported by our group [8] and also by others [15,16] that purslane could withstand salinity higher than 200 mM NaCl but it had better growth and higher productivity when grown under lower salinity conditions. The above results also supported that higher salinity such as 40% ASW contained 177 mM NaCl (calculation details seen in Section 3.1), which resulted in decreases in purslane shoot and root production compared to those grown with a lower salinity of 10% ASW containing 44 mM NaCl. In the previous study, purslane had been grown hydroponically in different NaCl salinities for 33 days before harvest. The values of shoot FW were about 15 g and 3 g, in 100 and 200 mM NaCl under LED R/B 2.2 at a photosynthetic photo flux density (PPFD) of 200 μmol m^−2^ s^−1^ (12 h), respectively [8]. To establish a more effective growth strategy, purslane plants were grown aeroponically using ASW under two different LED spectral qualities, R/B 0.9 and 2.2 at a PPFD of 330 μmol m^−2^ s^−1^ (12 h). Fourteen days after transplanting, shoot FWs of about 12 g and 5 g were recorded for purslane plants, in 10% ASW and 40% ASW, respectively (Figure 1A). The faster growth rate of purslane observed in this study could be due to the different growing systems and/or light intensities. We have also found that there were significant interactions between the LED spectra and salinity on shoot FW and DW of ice plants [21]. Giménez et al. [24] also reported that the yield of purslane microgreens was affected by both light quality and salinity. However, the influences of LED spectral quality and salinity effects on purslane shoot production were not found in this study. For the shoot/root FW ratio, purslane grown in 10% ASW under R/B 0.9 had the highest values followed by that grown in 10% ASW under R/B 2.2 and in 40% ASW under R/B 0.9, and that in 40% ASW under R/B 2.2 had the lowest value (Figure 2E). Statistically, all plants had a similar root DW (Figure 2D) and shoot/root DW ratio (Figure 2F). Higher root FW but not root DW of purslane grown under R/B 2.2 with 10% ASW conditions (Figure 2D) may be due to the water accumulation effect in roots, which may be associated with root morphology such as root length influenced by salinity [15,26,27,28] and/or LED spectral quality [29,30,31]. Kafi and Rahhimi [15] reported that salinity resulted in a decrease in root volume, surface area, diameter and total length. In the study with two other halophytes, *Glaux maritima* and *Spergularia marin*, it was reported that high salinities not only reduced the growth of both shoot and root, but also altered the morphological traits of shoot and roots [27]. Under salinity stress, plant roots had their own adaptive strategies in changing not only morphological traits such as root length and root surface area but also the anatomical structure such as the root xylem thickness and root phloem thickness [28]. Apart from the impact of salinity on root development, light moderates root growth and development [29]. For instance, blue light shortened the time required for root formation in sweet basil (*Ocimum basilicum* L.) [30]. Red light reduced the root FW and DW of tomato plants compared to white light, while BL did not cause any difference [31]. Thus, our future studies should include direct measurements of root morphological and anatomical traits.

**Figure 3:**
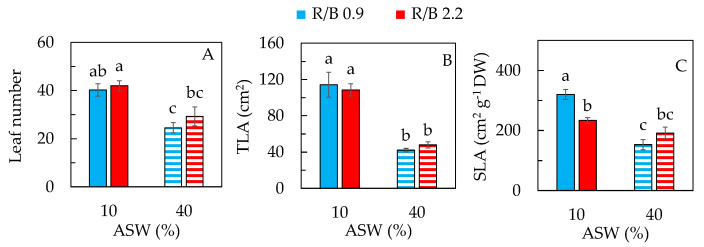
Leaf number (**A**); total leaf area, TLA (**B**); and specific leaf area, SLA (**C**), of purslane grown in two different percentages of artificial seawater (ASW) under two different LED R/B ratios for 14 days. Values are means ± standard error of 4 different plants. Means with different letters above the column represent significant differences according to Tukey’s multiple comparison test (*p* < 0.05).

Similar to the results of shoot FW (Figure 2A), purslane grown in 10% ASW had a greater total leaf number and larger total leaf area (TLA) than that in 40% ASW under both R/B ratios (Figure 3A,B). There were no significant differences in these two leaf traits between purslane under the two different R/B ratios in either 10% ASW or 40% ASW. Purslane grown in 10% ASW under R/B 0.9 had the highest specific leaf area (SLA) followed by that grown in 10% ASW and 40% ASW under R/B 2.2. The lowest SLA was observed in purslane grown in 40% ASW under R/B 0.9, resulting from the lowest TLA (Figure 3B) and the lowest leaf water content, LWC, but the highest leaf dry matter content, LDMC (Figure S2). After exposure to high salinity stress, cell water content of most plants generally decreases, which slows the rate of cell division and elongation [32]. In our previous study with aeroponically grown purslane under LED R/B 2.2 in different NaCl salinities, it was also found that lower SLA was associated with lower TLA and LWC but higher LDMC in higher salinities [8]. Similar correlations between SLA and TLA and between LWC and LDMC were also found in halophyte ice plants grown in different salinities [21].

Lower leaf number (Figure 3A) and smaller TLA (Figure 3B) resulted in lower shoot FW (Figure 2A) of purslane grown in higher salinity compared to that grown in lower salinity. The results suggest that shoot biomass accumulation mainly results from leaf initiation and expansion. Similar results were also found in our previous study with hydroponically grown purslane [8]. Under salinity stress conditions, decreases in leaf number, leaf area and growth rate were also observed in other halophytes [33] and vegetable crops [34,35]. This study also attempted to investigate the influence of light quality on the responses of purslane to salinity. However, LED spectral quality did not affect these two leaf traits grown under each of the two different salinities, which was different from the results of ice plants reported by our team [21]. Furthermore, purslane grown under LED R/B 0.9 in 10% ASW had the thinnest leaves. This was opposite to the results of ice plants, which had thinner leaves under a higher LED R/B ratio of 2.0 grown with a low salinity of 100 mM NaCl [21]. These results indicate that the interaction between LED quality and salinity on leaf traits is species-dependent.

### 2.2. Photosynthetic Pigments and Maximum Quantum Efficiency of PSII

**Figure 4:**
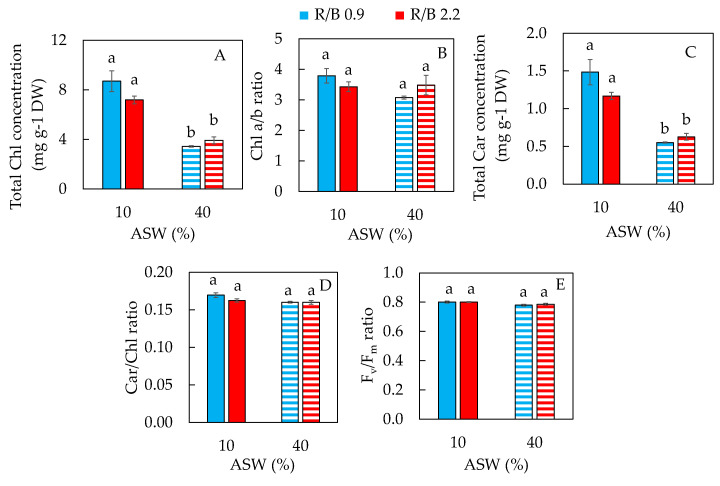
Total Chl concentration (**A**), Chl a/b ratio (**B**), total Car concentration (**C**), Car/Chl ratio (**D**) and F_v_/F_m_ ratio (**E**) of purslane grown in two different percentages of artificial seawater (ASW) under two different LED R/B ratios for 14 days. Values are means ± standard error of 4 different plants. Means with different letters above the column represent significant differences according to Tukey’s multiple comparison test (*p* < 0.05).

The total chlorophyll (Chl) (Figure 4A) and carotenoids (Car) concentrations (Figure 4C) were significantly higher in purslane grown in 10% ASW than in 40% ASW. Grown under high salinity conditions, reductions in total Chl and Car observed in this study may be due to increased degradation or reduced synthesis [36]. In our previous study with purslane, it was also found that purslane grown in a lower salinity such as 100 mM NaCl had higher total Chl and Car concentrations than that grown in higher salinities with 200 mM NaCl and 300 mM NaCl [8]. The higher total Chl and Car concentrations observed in the earlier study could be due to the two different cultivation methods and the ages of the leaves. On a DW basis, total Chl and Car concentrations of the two different studies were indeed of the same magnitude. However, in our previous study with halophyte ice plants grown under higher salinities with 250 and 500 mM NaCl, they had higher total Chl and Car concentrations compared to those grown with a lower salinity of 100 mM NaCl [21]. Similarly, in the study with two obligate halophytes, *Sesuvium portulacastrum* and *Tecticornia indica*, it was found that under saline conditions (200 mM and 400 mM NaCl), total Chl and Car concentrations were significantly enhanced in both species compared to non-saline conditions [37]. Ghanem et al. [33] reported that three obligate halophytes, namely, *Arthrocnemum macrostachyum*, *Sarcocornia fruticosa* and *Salicornia europaea*, rearranged their chlorophyll pigments after exposure to different salinities from 0 to 600 mM NaCl. When obligate halophyte ice plants were grown in lower salinity conditions with 100 mM and 250 mM NaCl, total Chl concentration under R/B 0.9 and 2.0 were higher than under R/B 2.8. However, ice plants grown with 500 mM NaCl showed the opposite results [21]. In our previous study with ice plants aeroponically grown in fresh water, all plants had similar Chl a/b and Chl/Car ratios under different combinations of R/B LED ratios [38]. These findings suggest that the effects of LED spectral quality on photosynthetic pigments were influenced by the salinity under which the ice plants were grown. In this study, there were no differences in Chl and Car concentrations in purslane grown under R/B 0.9 and R/B 2.2 for each of the two different saline conditions (Figure 4A,C). The Chl a/b ratio (Figure 4B) and Car/Chl ratio (Figure 4D) of purslane were also similar regardless of salinities and LED R/B ratios. However, in our previous study with purslane, higher salinity (300 mM NaCl) enhanced the Chl a/b ratio and Chl/Car ratio compared to those in purslane grown under lower salinity conditions [8]. In the study of two obligate halophytes, *S. portulacastrum* and *T. indica*, their Chl a/b ratios were higher under high salinity [37]. In the study with salt-tolerant *Populus talassica* × *Populus euphratica*, total Chl concentration and Chl a/b ratio decreased under 400 mM NaCl treatment compared to control plants grown under non-salinity conditions [28]. In ice plants, no clear trends were observed in Chl a/b and Chl/Car ratios among the different salinities and LED R/B ratios [21]. Although purslane grown with 40% ASW had a lower total Chl concentration than with 10% ASW (Figure 4A), there was no significant difference in F_v_/F_m_ ratios which were close to 0.8 (Figure 4E). This result indicates that there was no evidence of damage to PS II [39,40]. In the study with purslane by the other group, it was reported that F_v_/F_m_ ratios remained unchanged after exposure to 100 mM and 300 mM NaCl for 9 days [40]. Similar results were also obtained in our previous study with purslane [8] and ice plants [21]. Summing up, the impacts of salinity and LED spectral quality on photosynthetic pigments and light use efficiency depend on both the experimental conditions and species.

### 2.3. Nitrogen Metabolism

**Figure 5:**
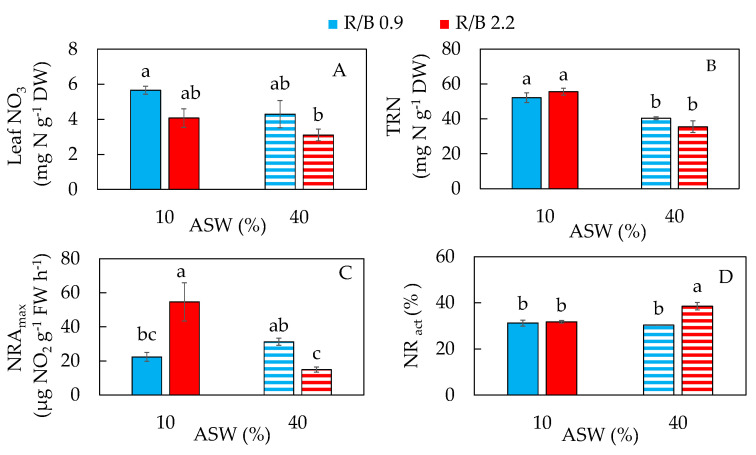
Leaf NO_3_^−^ concentration (**A**); leaf total reduced nitrogen, TRN, concentration (**B**); leaf maximum nitrate reductase activity, NRA_max_ (**C**); and nitrate reductase activation state, NR_act_ (**D**), of purslane grown in two different percentages of artificial seawater (ASW) under two different LED red/blue ratios for 14 days. Values are means ± standard error of 3 (**A**,**B**) or 4 (**C**,**D**) replicates. Means with different letters above the column represent significant differences according to Tukey’s multiple comparison test (*p* < 0.05).

Salinity was reported to affect N metabolism including NO_3_^−^ uptake and NO_3_^−^ assimilation by disturbing the activities of the main enzymes involved in nitrogen metabolism such as NR [41]. Leaf NO_3_^−^ concentration was the highest in purslane grown in 10% ASW under R/B 0.9, which was significantly higher than that of purslane in 40% ASW under R/B 2.2. Grown in 10% ASW under R/B 2.2 and in 40% ASW under R/B 0.9, leaf NO_3_^−^ concentrations of purslane were statistically similar to the other two treatments (Figure 5A). No significant difference in TRN was observed between plants grown under both R/B ratios in either 10% AWS or 40% ASW (Figure 5B). Although purslane is a NO_3_^−^-accumulating plant, its NO_3_^−^ concentration depends on growing conditions [42]. In our previous study, leaf NO_3_^−^ concentration was about twofold higher in the hydroponically grown purslane with 0 and 100 mM NaCl than with 200 and 300 mM NaCl [8]. The highest leaf NO_3_^−^ concentration for aeroponically grown purslane in this study was about 6 mg N g^−1^ DW (Figure 5A), which was much lower compared to the highest concentration of >15 mg N g^−1^ DW in the hydroponically grown purslane in our previous study [8]. After conversion, the highest NO_3_^−^ concentration of purslane grown hydroponically was 1235 mg kg^−1^ FW, which was much lower than 2500 mg kg^−1^ FW as reported by Corrè and Breimer [43]. With less NO_3_^−^ accumulated, together with an adequate amount of shoot TRN that is greater than 2% (Figure 5B), this contributes to the quality of aeroponically grown purslane in this study.

Purslane grown in 10% ASW under R/B 2.2 had the highest NRA_max_. However, there was no significant difference in NRA_max_ between purslane grown in 10% ASW under R/B 2.2 and in 40% ASW under R/B 0.9 and between 10% ASW under R/B 0.9 and 40% ASW under R/B 2.2 (Figure 5C). NR_act_ was significantly higher in 40% ASW under R/B 2.2 compared to those of the other three conditions which had similar NR_act_ (Figure 5D). These results suggest that there were no clear trends in the responses of NRA_max_ to salinity and LED spectral quality. There was a negative correlation between NRA_max_ and NR_act_ for purslane grown in 40% ASW under LED R/B 2.2, which had the lowest NRA_max_ but the highest NR_act_. However, for purslane grown under other conditions, NRA_max_ was not correlated with NR_act_ (Figure 5C,D). When hydroponically grown maize seedlings (*Zea mays* L.) were exposed to moderate salinity, NRA_max_ and NR-mRNA were lower under salinity conditions, but there were no changes in the NR_act_ compared to those grown under non-saline conditions [44]. In the study with *Thellungiella halophila*, a salt-tolerant relative of Arabidopsis thaliana, it was found that the NRA increased significantly with increasing salinity [45]. In our recent study, it was found that NRA_max_ was inhibited with full nitrogen (NO_3_^−^) supply in a C_3_-CAM halophyte ice plant [12]. Impacts of NO_3_^−^ supply on a C_4_-CAM purslane grown in different salinities merit our further study.

### 2.4. Phytochemical, Antioxidant and Antioxidant Enzyme Activities

**Figure 6:**
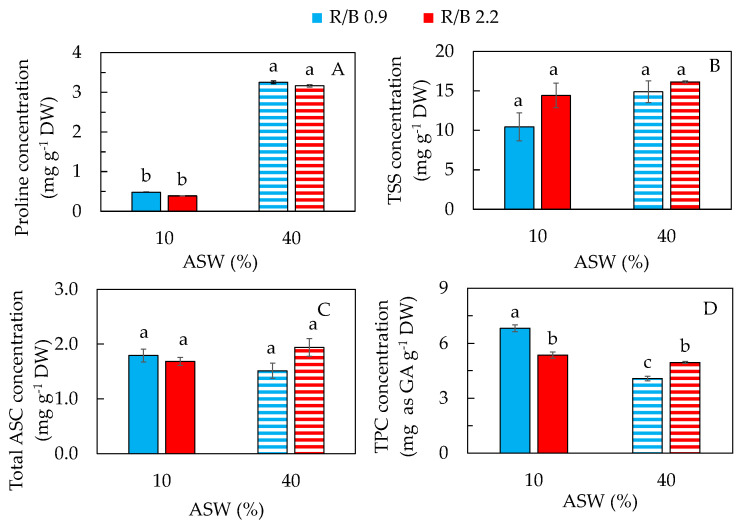
Concentrations of proline (**A**); total soluble sugar, TSS (**B**); total ascorbic acid, ASC (**C**); and total phenolic compounds, TPC (**D**), of purslane grown in two different percentages of artificial seawater (ASW) under two different LED R/B ratios for 14 days. Values are means ± standard error of 3 (**B**,**D**) or 4 (**A**,**C**) replicates. Means with different letters above the column represent significant differences according to Tukey’s multiple comparison test (*p* < 0.05).

Proline is the most common endogenous osmolyte accumulated under various abiotic stresses. Under salinity stress, apart from acting as an osmolyte for osmotic adjustment, proline also enhances the capacity of the antioxidative system to scavenge ROS [46]. In this study, proline concentration was about sevenfold higher in purslane grown in 40% ASW than in 10% ASW under both R/B ratios. There was no significant difference in proline concentration between the two different R/B ratios under the same percentage of ASW (Figure 6A). In our previous study, it was also found that purslane grown in 300 mM NaCl had the highest leaf proline concentration compared to that grown under lower NaCl concentrations [8]. The proline concentration (~3.2 mg g^−1^ DW) of purslane grown in 40% ASW containing 177 mM NaCl under both R/B ratios was much lower compared to that of purslanes grown in 200 mM NaCl (~20 mg g^−1^ DW) in our previous study [8]. The difference in proline concentrations between the two studies could be due to the different cultivation methods and/or the leaf ages of 14 and 33 days for this and the previous study, respectively. In another two studies by other teams, it has also been reported that high salinity enhanced proline concentration in purslane [19,40]. In the study with three halophytes such as *A. macrostachyum*, *S. fruticosa* and *S. europaea* from the same ecological habitat, it was found that they engaged two different salt-tolerant strategies [33]. *S. europaea* activated antioxidant enzymes and the biosynthesis of proline. This was supported by the fact that biomass decreased with increased proline concentrations and peroxidase activity when grown in high salinity. However, for *A. macrostachyum* and *S. fruticose*, the proline concentration was decreased while the Chl a/b ratio and antioxidant compounds were increased. It was concluded that the salt tolerance mechanism of halophytes is species-specific.

Apart from proline, the accumulation of TSS also plays an important role in osmotic adjustment under salinity stress [21,28,47,48,49,50]. In this study, however, there were no significant differences in TSS concentration (Figure 6B) among the different treatments. This result is similar to our previous study with purslane, which had similar TSS concentrations in the leaves regardless of the salinity conditions [8]. In contrast, salinity stress enhanced the accumulation of TSS in the leaves of ice plants [21] and *T. halophila* [50]. After 45 days of treatment, the soluble sugar concentration of *P. talassica* × *P. euphratica* leaves decreased under 200 mM NaCl but increased under 400 mM NaCl treatments compared to the control plant [28]. Grown under 120 mM NaCl conditions for 20 days, a salt-tolerant asparagus cultivar had an increased TSS concentration, while this was not found in a salt-sensitive asparagus cultivar [49]. Thus, the accumulation of soluble sugar depends on the intensity of salinity stress [28], cultivar and leaf age [51].

There are limited studies available on the effects of salinity on the accumulation of ASC and TPC in purslane under different LED spectral qualities. In this study, all purslane plants had similar levels of ASC (Figure 6C). In our previous study, ASC concentration in purslane grown hydroponically with 100 mM NaCl was higher than that grown with 0 mM, 200 mM and 300 mM NaCl [8]. These results indicate that low salinity enhances ASC accumulation in purslane compared to that grown with fresh water. However, increasing NaCl concentration from 100 mM to 200 or 300 mM in the previous study [8] or increasing ASW from 10% to 40% in this study did not increase the ASC concentration. Furthermore, LED spectral quality did not influence the salinity effects on ASC production. Similar to the leaf proline concentration (Figure 6A), purslane grown in 40% ASW containing 177 mM NaCl under both R/B ratios had a much lower ASC concentration (1.5–2 mg g^−1^ DW) compared to that of purslane grown under 200 mM NaCl (~4 mg g^−1^ DW) [8]. As mentioned earlier, different cultivation methods and/or the leaf ages may be responsible for the different concentration.

It is well known that biochemical changes have been observed to help halophytic plants survive under salinity conditions [33]. Accumulation of non-enzymatic antioxidants such as phenolic compounds is one of the adaptive strategies to salinity [52,53]. According to Uddin et al. [54], the antioxidant potential of purslane mainly depends on the accumulation of total phenolic compounds (TPC). In this study, the TPC concentration was the highest in purslane grown in 10% ASW under R/B 0.9 followed by that grown in 10% ASW and 40% ASW under R/B 2.2. Plants grown in 40% ASW under R/B 0.9 had the lowest TPC concentration (Figure 6D). In the study of three different halophytes from the same ecological habitat, TPC concentration in *A. macrostachyum* increased significantly at 200 and 400 mM NaCl, but there was no significant difference at 100 mM NaCl compared to 0 mM NaCl. For *S. fruticosa* and *S. europaea* plants, they had similar levels of TPC under all salinity conditions. However, all three species showed significant decreases in TPC at 600 mM NaCl [33]. Similar to our previous result [8] and the other study by the other team with purslane [55] and other halophytes [33], a great variation in the accumulation of TPC directly depends on genetics and other growth conditions.

**Figure 7:**
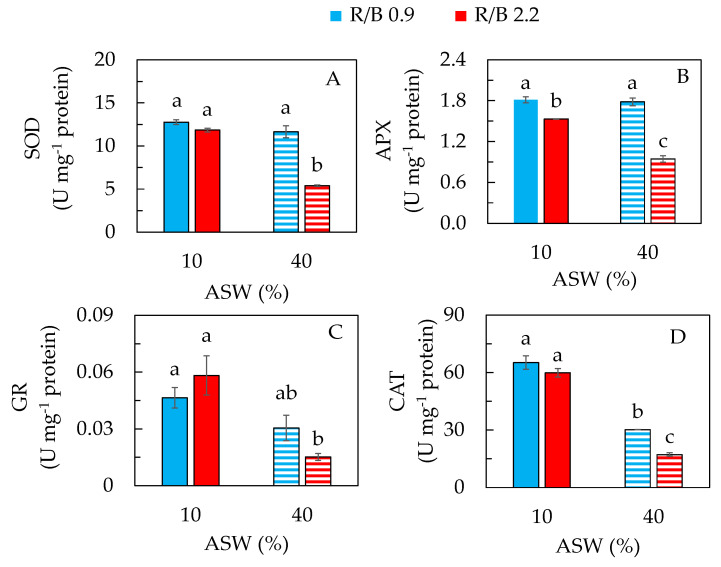
Activities of superoxide dismutase, SOD (**A**); ascorbate peroxidase, APX (**B**); glutathione reductase, GR (**C**); and catalase, CAT (**D**), of purslane grown in two different percentages of artificial seawater (ASW) under two different LED R/B ratios for 14 days. Values are means ± standard error of 4 replicates. Means with different letters above the column represent significant differences according to Tukey’s multiple comparison test (*p* < 0.05).

Mechanisms for plants to protect against salt stress also include enzymatic molecules with antioxidant function. The antioxidant enzymes mainly consist of SOD, APX, GR and CAT [33,49,56]. Thus, this study also aimed to investigate if the activities of the antioxidant enzymes in purslane grown under different LED spectral qualities are correlated with non-enzymatic antioxidants such as proline. Overall, purslane grown in 40% ASW under R/B 2.2 had significantly lower activities of SOD (Figure 7A), APX (Figure 7B), GR (Figure 7C) and CAT (Figure 7D) compared to the other three conditions. There were no significant differences in SOD and GR activities among purslane grown in 10% ASW under both R/B ratios and in 40% ASW under R/B 0.9 (Figure 7A,C). Purslane grown in 10% ASW under both R/B ratios and in 40% ASW under R/B 0.9 had significantly higher APX activity than that of plants grown in 40% ASW under R/B 2.2 (Figure 7B). Purslane grown in 10% ASW under both R/B ratios similarly had higher CAT activity than purslane grown in 40% ASW under R/B 0.9 (Figure 7D). These results suggest that high salinity such as 40% ASW decreased the activities of antioxidant enzymes such as SOD, APX and GR under LED R/B 2.2 and the activity of CAT under both R/B ratios. However, in this study, when grown under R/B 0.9, there were no significant differences in the activity of SOD, APX and GR between 10% ASW and 40% ASW. In response to salt stress, salt-tolerant species increased the activities of antioxidant enzymes, while salt-sensitive species were unable to perform the same [57]. Purslane is considered a moderately salt-tolerant species [8], and it decreased the activities of antioxidant enzymes when grown with 40% ASW containing 177 mM NaCl under R/B 2.2 with enhanced proline concentration (Figure 6A). In the other study by the other team, it was found that purslane treated with 140 mM NaCl for 18 days had lower activities of SOD and APX but high activities of GR and CAT and proline concentration compared to the control plants grown without NaCl [19]. In the study with asparagus, activities of SOD and CAT gradually increased under salt stress in a salt-tolerant cultivar, while the corresponding enzyme activities in a salt-sensitive cultivar were decreased after 20 days of growth under 120 mM NaCl conditions [49]. These findings suggest that the impacts of salinity on antioxidant enzymes and the correlation between antioxidant enzymes and antioxidants depend on species and environmental conditions.

## 3. Materials and Methods

### 3.1. Plant Materials

**Figure 8:**
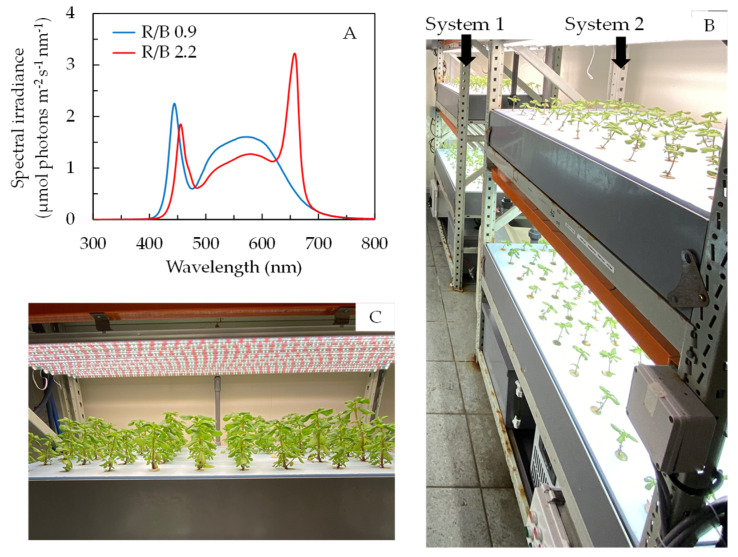
(**A**) LED spectra of R/B 0.9 and R/B 2.2. Spectral scans were recorded every 0.5 nm with a spectroradiometer (PS300, Apogee Instruments, Logan, Utah, USA). (**B**) Vertical aeroponic systems with purslane plants and (**C**) purslane plants grown in 10% ASW on indoor vertical aeroponic systems.

Seeds of purslane (*P. oleracea* L. cv. POR—2936) were germinated on moist filter paper in petri dishes and were incubated under a PPFD of an average of 275 µmol m^−2^ s^−1^ provided by an LED (WR-16W, Beijing Lighting Valley Technology Co., Ltd., Beijing, China) with a red/blue (R/B) ratio of 2.2 for a 12 h photoperiod from 9:00 to 21:00 daily. The LED spectrum of R/B 2.2 is shown in Figure 8A. After 3 days, purslane seedlings were then inserted into polyurethane cubes and grown in plastic trays filled with modified full-strength Netherlands Standard Solution (2.2 ± 0.2 mS cm^−1^ conductivity and pH 6.0 ± 0.2) described in He et al. [58]. All plants were placed under R/B 2.2 with a PPFD of 330 ± 30 µmol m^−2^ s^−1^ for 12 h from 9:00 to 21:00 daily for 4 weeks before transplanting onto the two different vertical aeroponic systems in an air-conditioned room (Figure 8B). The size of each aeroponic trough was 120 cm × 60 cm × 14 cm (length × width × height). There were 60 plants planted on the top of each trough. The plant density was about 80 m^−2^. The capacity of the nutrient tank for each system was 40 L. Purslane plants were grown in 10% ASW (System 1, Figure 8B) and 40% ASW (System 2, Figure 8B) with modified full-strength Netherlands Standard Solution [59] under R/B 0.9 (lower level) and R/B 2.2 (upper level) with PPFDs of 330 ± 30 µmol m^−2^ s^−1^ (Figure 8A). The conductivity and pH were 2.2 ± 0.2 mS cm^−1^ and 6.0 ± 0.2, respectively, for the modified full-strength Netherlands Standard Solution in both aeroponic nutrition tanks. All plants were grown for 14 days before harvest. Figure 8C shows purslane plants grown in 10% ASW under R/B 2.2. In this study, 100% ASW with a salinity of 33 ppt contains 36 g of Red Sea Salt^®^ (Red Sea Fish Pharm Ltd., Eilat, Israel; www.redseafish.com, accessed on 10 July 2023) in 1 L water. The two different salinity conditions, i.e., 10% and 40% ASW, were obtained by dissolving 3.6 g and 14.4 g of Red Sea Salt^®^ per liter in modified full-strength Netherlands Standard Solution, respectively. According to the Red Sea Salt advanced formula, naturally harvested salt from the Red Sea comprises 72% of the salt mix, which includes 45 minor and trace elements. Other major, minor and trace elements comprise 28% of the salt mix. NaCl molarity in ASW has been calculated as follows:

100% ASW = 36 g Red Sea salt/L

NaCl MW is 58.44 g/mol. Red Sea salt comprises 72% NaCl.

100%ASW = 36 × 72%/58.44 = 0.44353 M = 443.53 mM

10% ASW = 443.53/10 = 44.353 mM

40%ASW = 44.353 × 4 = 177.412 mM

The duration of the nutrient spray was 30 s at 5 min intervals. During the 30 s nutrient spay period, nutrient solution was uniformly sprayed across each trough. The aeroponic fertigation system was described in more detail by He in 2010 [59]. The temperature and relative humidity in the air-conditioned room ranged from 20.5 °C to 23.5 °C and 53% to 92%, respectively.

### 3.2. Measurement of Shoot and Root Productivity and Leaf Traits

Fourteen days after transplanting, four purslane plants were randomly harvested, removing the polyurethane cubes. The number of leaves were counted. TLA was obtained using a leaf area meter (WinDIAS3 Image Analysis system). The leaves, stems and roots were weighed to obtain the FW before drying them at 80 °C for 4 days. The different plant parts together with the aluminum foil were reweighed to obtain the DW. The measurements of SLA were obtained as follows: SLA = leaf Area (cm^2^)/DW (g).

### 3.3. Measurement of Photosynthetic Pigments

Fourteen days after transplanting, one disc (1 cm in diameter) of the youngest fully expanded leaves was first punched using a cork borer from each of the four randomly harvested plants (n = 4). The respective fresh weight of a leaf disc was recorded before being placed in 5 mL of N, N-dimethylformamide (Sigma Chemical Co., Darmstadt, Germany) in darkness for 48 h at 4 °C. Each supernatant was decanted into a cuvette and a spectrophotometer (Shimadzu UV-1800 UV-Vis spectrophotometer, Kyoto, Japan) was used to determine the absorptions of different pigments based on Welburn [60].

### 3.4. Measurement of Chl Fluorescence F_v_/F_m_ Ratio

Fourteen days after transplanting, the four youngest fully expanded attached leaves from four randomly selected plants were adapted to the dark for 15 min mid-photoperiod before placing under the light pipe of the Plant Efficiency Analyzer, PEA (Hansatech Instruments Ltd., Norfolk, UK). The initial fluorescence (F_o_) was measured before obtaining the maximum fluorescence (F_m_) using 0.8 s of a saturated pulse. Variable fluorescence, F_v_, was calculated as F_v_ = F_m_ − F_o_.

### 3.5. Measurements of Leaf NO_3_^−^ and TRN

The procedure of leaf NO_3_^−^ measurement was based on He et al. [59]. Dried leaf samples (0.01 g) were ground with 10 mL Milli-Q water. The mixture was shaken at 37 °C, 200 rpm for 2 h. The mixture was then filtered through a 0.45 μm pore membrane using a vacuum filter. The filtrate was topped up to 50 mL with acidified Milli-Q water. The NO_3_^−^ concentration was determined using the Flow Injection Analyzer (Model Quikchem 8000, Lachat Instruments Inc., Milwaukee, WI, USA). The resulting magenta solution was read at 520 nm to obtain the NO_3_^−^ concentration. To determine TRN concentration, 0.05 g of dried samples was digested with a Kjeldahl tablet in 5 mL of concentrated sulfuric acid for 60 min at 350 °C. After digestion, a Kjeltec 8400 analyzer (Foss Tecator AB, Höganäs, Sweden) was used to quantify TRN concentration.

### 3.6. Measurements of NRA_max_ and NR_act_

The frozen sample was powdered in liquid nitrogen before being ground in ice-cold buffer (0.25 M Tris-HCl (pH 8.5), 3 mM dithiothreitol (DTT), 10 µM flavin adenine dinucleotide (FAD), 1 µM sodium molybdate, 1 mM Ethylenediamine-tetra-aceticacid (EDTA)) based on Kuo et al. [61] with the presence of PVPP. After centrifuging the extract at 18,000 g for 20 min at 4 °C, the supernatant was used immediately according to Kaiser and Huber [62] with modification. The NR_act_ was determined by assaying NR either with Mg^2+^ (10 mM) or with EDTA (15 mM) in the reaction medium (50 mM Hepes-KOH (pH 7.5), 1 mM DTT, 10 µM FAD, 10 mM KNO_3_, 0.2 mM NADH, NR extraction and 10 mM MgCl_2_ or 15 mM EDTA). Incubation was performed at 25 °C for 20 min, and the reaction was terminated by Sulfanilamide (1%(*w*/*v*) in 3 N HCl) and naphthylethylens-diamine dihydrochloride (0.02% *w*/*v*). The absorption of each sample was read at A_540nm_. For the blank, NR extracts were heated at 80 °C for 5 min before being added to the same reaction solution described above. NR_max_ was calculated as µg NO_2_^−^ h^−1^ g^−1^ FW. The NR_act_ is the activity measured in the presence of 10 mM MgCl_2_ divided by the activity measured in the presence of 15 mM EDTA.

### 3.7. Determination of TSS Concentration

Dried leaf samples (0.01 g), were added to 4 mL of 80% ethanol before heating for 30 min in a 65 °C water bath. The supernatant was collected after centrifuging at 3500 rpm for 5 min. The pellet was resuspended in an additional 2 mL of 80% ethanol twice. The concentration of free soluble sugar was determined at 490 nm [63].

### 3.8. Determinations of Proline, Total ASC and TPC Concentrations

The same amount of frozen plant tissues (0.5 g) was used to extract proline, ASC and TPC separately. The details of extraction for each of the three phytochemicals were described by He et al. [58]. The proline assay was modified based on Bates et al. [64]. The absorbance of the proline extract was measured at 520 nm. For ASC and TPC, the absorbances were determined at 524 nm and 765 nm, respectively, according to Leipner et al. [65] and Ragee et al. [66]. All absorbances were measured using a spectrophotometer (Shimadzu UV-1800 UV-Vis spectrophotometer, Kyoto, Japan).

### 3.9. Determination of Activities of Antioxidant Enzymes

Frozen leaf tissues (0.5 g) were ground in liquid nitrogen and homogenized in 4 mL ice-cold extraction buffer (50 mM potassium phosphate buffer, pH 7.0, 1 mM EDTA-Na_2_ and 5% (*w*/*v*) polyvinylpolypyrrolidone). the homogenate was centrifuged at 18,000× *g* for 20 min at 4 °C. The supernatants were used for the assays of SOD, GR and CAT. Extraction media for APX were prepared with inclusion of ascorbic acid (5 mM) in the main extraction buffer to protect the APX activity.

Total SOD (EC 1.15.1.1) activity was determined by measuring its ability to inhibit the photochemical reduction of nitro blue tetrazolium chloride (NBT) based on Droillard et al. [67] and modified by Sarker and Oba [68]. An amount of 0.1 mL of 2µM riboflavin was added in 2.9 mL of the reaction mixture containing 13.33 mM methionine, 75 µM NBT, 0.1 mM EDTA-Na_2_, 50 mM phosphate buffer (pH 7.0), 50 mM sodium carbonate and 0.1 mL of enzyme extract. Tubes were shaken and illuminated for 10 min under 300 μmol m^−2^ s^−1^ irradiance. After the incubation, the test tubes and rack were covered with aluminum foil. The absorbance was recorded immediately at 560 nm in the dark using a spectrophotometer (Shimadzu UV-1800 UV-Vis spectrophotometer, Kyoto, Japan). The reaction mixture with no enzymes developed the maximum color due to the maximum rate of reduction of NBT. Enzyme activity was calculated as the amount of enzyme that reduced the absorbance reading to 50% in comparison with tubes lacking enzymes.

Ascorbate peroxidase (APX, EC 1.11.1.11) activity was determined based on Nakano and Asada [69] modified by Rangani et al. [70] and Saikachout et al. [71], depending on the decrease in absorbance at 290 nm as ascorbate was oxidized. The reaction mixture (3 mL) contained 50 mM potassium phosphate buffer (pH 7.0), 1 mM EDTA-Na2, 0.5 mM ascorbic acid, 0.1 mM H_2_O_2_ and enzyme extract (50 μL). The reaction was initiated by the addition of H_2_O_2_. The decrease in the absorption at 290 nm was recorded at an interval of 10 s for 1 min with a spectrophotometer (Shimadzu UV-1800 UV-Vis spectrophotometer, Kyoto, Japan). APX activity was calculated using an extinction coefficient at 290 nm of 2.8 mM^−1^ cm^−1^ for ascorbate. An amount of 1 μmol ascorbate oxidized per minute was defined as one unit of APX.

Glutathione reductase (GR, EC 1.6.4.2) activity was determined using a Glutathione Reductase Assay Kit (Catalog Number: GRSA, Sigma-Aldrich, Saint Louis, USA). GR activity was measured by the decrease in absorbance at 340 nm caused by the oxidation of NADPH using an extinction coefficient of 6.22 mM^−1^ cm^−1^ for NADPH at 25 °C. The UV assay medium contained 500 µL of 2 mM oxidized glutathione (GSSG), 50 µL of 2 mM NADPH, 150 µL of Glutathione Reductase Assay Buffer (100 mM potassium phosphate buffer, pH 7.5, with 1 mM EDTA) and 300 µL of enzyme extract. The decrease in the absorption at 340 nm was recorded at an interval of 10 s for 110 s with a spectrophotometer (Shimadzu UV-1800 UV-Vis spectrophotometer, Kyoto, Japan). An amount of 1 μmol NADPH oxidized per minute was defined as one unit of GR.

Catalase (CAT; EC 1.11.1.6) activity was determined according to Aebi [72] modified by Rangani et al. [70] and Saikachout et al. [71], which measures the initial linear rate of degradation of H_2_O_2_ at 240 nm. The reaction mixture (3 mL) contained 50 mM potassium phosphate buffer (pH 7.0), 15 mM H_2_O_2_ and enzyme extract (100 μL) at 25 °C. The reaction was initiated by the addition of H_2_O_2_. The decrease in the absorption at 240 nm was recorded at an interval of 10 s for 1 min with a spectrophotometer (Shimadzu UV-1800 UV-Vis spectrophotometer, Kyoto, Japan). CAT activity was calculated using an extinction coefficient at 240 nm of 43.6 M^−1^ cm^−1^ for H_2_O_2_ [50]. An amount of 1 μmol H_2_O_2_ reduced per minute was defined as one unit of CAT.

### 3.10. Statistical Analysis

Data analysis was carried out using SPSS statistics 26.0 software and one-way ANOVA was used to determine the significant differences among treatments. Tukey’s multiple comparison tests were used to discriminate the means. When the *p* value was <0.05, it was considered significantly different.

## 4. Conclusions

We have previously studied the effects of different NaCl salinities on purslane grown hydroponically under an LED R/B ratio of 2.2 at a PPFD at 200 μmol m^−2^ s^−1^ (12 h). It was found that high NaCl salinity reduces plant growth and photosynthetic performance but enhances certain nutritional qualities [8]. To enhance productivity and nutritional quality of purslane grown indoors, the current research attempted to establish a more effective growth strategy. This study also investigated the influences of LED spectral quality on the artificial seawater (ASW) salinity effects of aeroponically grown purslane growth, physiology and nutritional quality. LED spectral quality influenced purslane root growth in 10% ASW, under which a higher percentage of R-LED (R/B 2.2) seemed to promote root development and water accumulation compared to that grown under a higher percentage of B-LED (R/B 0.9). The influence of LED spectral quality on shoot production was not found in purslane grown in either 10% or 40% ASW. There were no clear trends in the responses of leaf traits to LED spectral quality under each of the two different salinities. High salinity reduced total Chl and Car concentrations of purslane similarly under both R/B ratios. However, all plants had a similar Chl a/b ratio, Chl/Car ratio and F_v_/F_m_ ratio regardless of salinity and LED spectral quality. Although all purslane accumulated much less NO_3_^-^ compared to our previous study [8], it had adequate amounts of shoot TRN. Purslane grown in 10% ASW had the highest NRA_max_ under R/B 2.2, while NR_act_ was the highest for that grown with 40% ASW under R/B 2.2. It was found that purslane grown in 40% ASW had a sevenfold increase in proline concentration than that in 10% ASW. However, there were no clear correlations between proline and the activities of the antioxidant enzymes. The results of this study revealed a highly complex picture of N metabolism and nutritional quality of purslane grown in different percentages of ASW under different LED spectral qualities as there were no clear trends in their responses to the different treatments. Our recent study with purslane showed that increasing the daily light integral through the optimum combination of light intensity and photoperiod displayed positive effects on the productivity and nutritional quality of purslane [73]. The influences of LED spectral quality and salinity effects on purslane may also depend on the light intensity or daily light integral, which merit further study.

## Data Availability

The data are available on reasonable request in writing to the corresponding author.

## Supplementary Materials

The following supporting information can be downloaded at https://www.mdpi.com/article/10.3390/plants12183214/s1, Figure S1: Leaf FW (A), leaf DW (B), stem FW (C), stem DW (D) of purslane grown in two different percentages of artificial seawater (ASW) under two different LED R/B ratios for 14 days. Values are means± standard error of 4 different plants. Means with different letters above the column represent significant differences according to Tukey’s multiple comparison test (*p* < 0.05). Figure S2: Leaf water content, LWC (A) and leaf dry matter content, LDMC (B) of purslane grown in two different percentages of artificial seawater (ASW) under two different LED R/B ratios for 14 days. Values are means ± standard error of 4 different plants. Means with different letters above the column represent significant differences according to Tukey’s multiple comparison test (*p* < 0.05).
