# Supplementary material for: Growth, Physiology and Nutritional Quality of C4 Halophyte Portulaca oleracea L. Grown Aeroponically in Different Percentages of Artificial Seawater under Different Light-Emitting Diode Spectral Qualities
Source: Plants (Basel). 2023 Sep 8;12(18):3214. doi: 10.3390/plants12183214 (PMC10535323; doi:10.3390/plants12183214)
Supplement: Supplementary file 1 [file plants-12-03214-s001.zip › plants-2527251-supplementary.pdf]

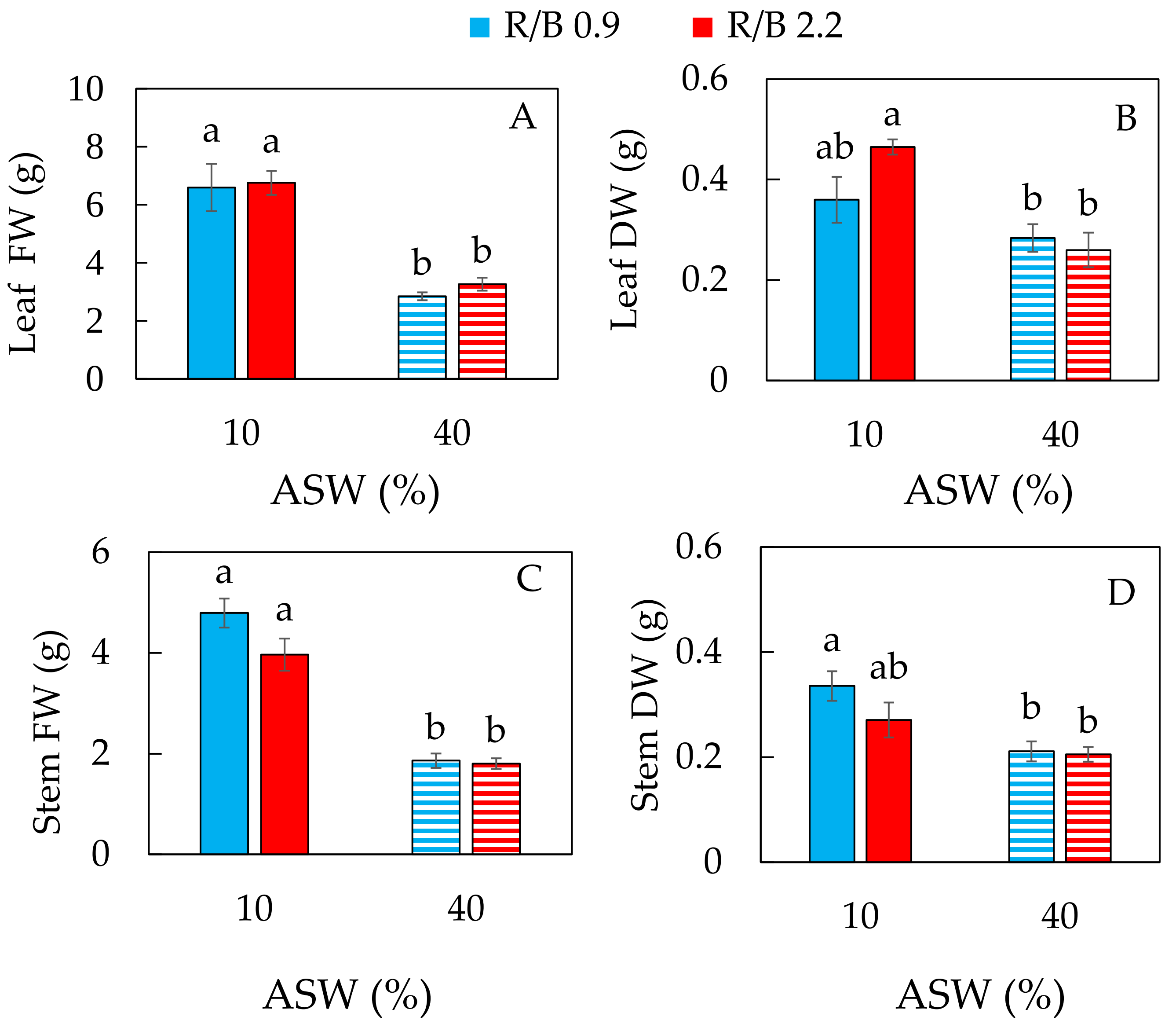

Figure 1S. Leaf FW (A), leaf DW (B), stem FW (C), stem DW (D) of purslane grown in two different percentages of artificial seawater (ASW) under two different LED R/B ratios for 14 days. Values are means ± standard error of 4 different plants. Means with different letters above the column represent significant differences according to Tukey's multiple comparison test (p<0.05).

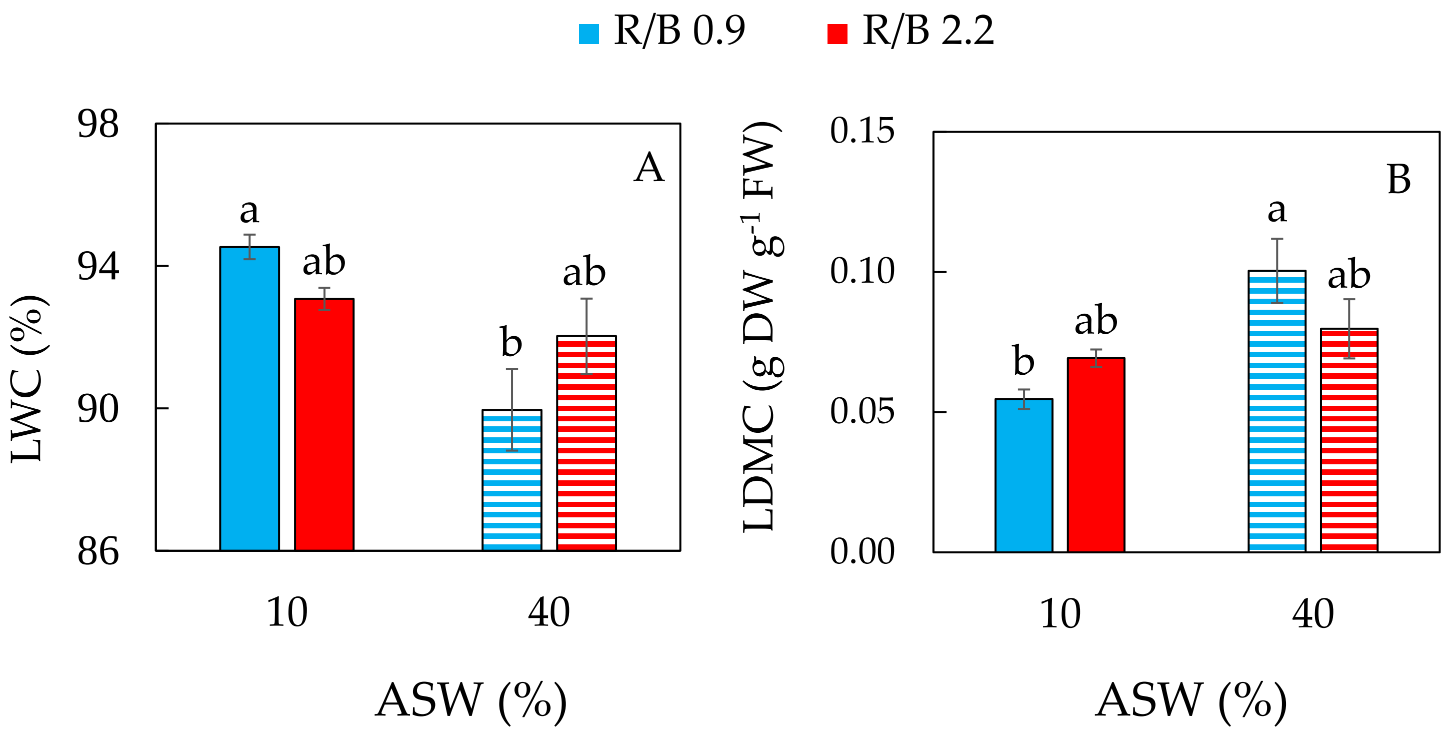

Figure 2S. Leaf water content, LWC (A) and leaf dry matter content, LDMC (B) of purslane grown in two different percentages of artificial seawater (ASW) under two different LED R/B ratios for 14 days. Values are means ± standard error of 4 different plants. Means with different letters above the column represent significant differences according to Tukey's multiple comparison test (p<0.05).
